# Emulating causal dose-response relations between air pollutants and mortality in the Medicare population

**DOI:** 10.1186/s12940-021-00742-x

**Published:** 2021-05-06

**Authors:** Yaguang Wei, Mahdieh Danesh Yazdi, Qian Di, Weeberb J. Requia, Francesca Dominici, Antonella Zanobetti, Joel Schwartz

**Affiliations:** 1 Department of Environmental Health, Harvard T. H. Chan School of Public Health, Landmark Center 4th West, 401 Park Drive, Boston, MA 02215 USA; 2 Vanke School of Public Health, Tsinghua University, Beijing, China; 3 School of Public Policy and Government, Fundação Getúlio Vargas, Brasília, Distrito Federal Brazil; 4 Department of Biostatistics, Harvard T. H. Chan School of Public Health, Boston, MA USA; 5 Department of Epidemiology, Harvard T. H. Chan School of Public Health, Boston, MA USA

**Keywords:** Air pollution, Chronic exposures, Mortality, Causal modeling, Does-response relations

## Abstract

**Background:**

Fine particulate matter (PM_2.5_), ozone (O_3_), and nitrogen dioxide (NO_2_) are major air pollutants that pose considerable threats to human health. However, what has been mostly missing in air pollution epidemiology is causal dose-response (D-R) relations between those exposures and mortality. Such causal D-R relations can provide profound implications in predicting health impact at a target level of air pollution concentration.

**Methods:**

Using national Medicare cohort during 2000–2016, we simultaneously emulated causal D-R relations between chronic exposures to fine particulate matter (PM_2.5_), ozone (O_3_), and nitrogen dioxide (NO_2_) and all-cause mortality. To relax the contentious assumptions of inverse probability weighting for continuous exposures, including distributional form of the exposure and heteroscedasticity, we proposed a decile binning approach which divided each exposure into ten equal-sized groups by deciles, treated the lowest decile group as reference, and estimated the effects for the other groups. Binning continuous exposures also makes the inverse probability weights robust against outliers.

**Results:**

Assuming the causal framework was valid, we found that higher levels of PM_2.5_, O_3_, and NO_2_ were causally associated with greater risk of mortality and that PM_2.5_ posed the greatest risk. For PM_2.5_, the relative risk (RR) of mortality monotonically increased from the 2nd (RR, 1.022; 95% confidence interval [CI], 1.018–1.025) to the 10th decile group (RR, 1.207; 95% CI, 1.203–1.210); for O_3_, the RR increased from the 2nd (RR, 1.050; 95% CI, 1.047–1.053) to the 9th decile group (RR, 1.107; 95% CI, 1.104–1.110); for NO_2_, the DR curve wiggled at low levels and started rising from the 6th (RR, 1.005; 95% CI, 1.002–1.018) till the highest decile group (RR, 1.024; 95% CI, 1.021–1.027).

**Conclusions:**

This study provided more robust evidence of the causal relations between air pollution exposures and mortality. The emulated causal D-R relations provided significant implications for reviewing the national air quality standards, as they inferred the number of potential early deaths prevented if air pollutants were reduced to specific levels; for example, lowering each air pollutant concentration from the 70th to 60th percentiles would prevent 65,935 early deaths per year.

**Supplementary Information:**

The online version contains supplementary material available at 10.1186/s12940-021-00742-x.

## Introduction

Fine particulate matter (PM_2.5_), ozone (O_3_), and nitrogen dioxide (NO_2_) are major air pollutants that pose considerable threats to human health [1, 2]. Starting in the 1990s, a large literature of epidemiological research has reported associations between chronic air pollution exposures and mortality, with PM_2.5_ and O_3_ being the most extensively studied components [3–9]. Chronic exposure to NO_2_ has also been associated with mortality, although the evidence is relatively scarce [10, 11]. These findings provide important implications for understanding the health burden attributable to poor air quality. In the United States, it is estimated that each 1 μg·m^− 3^ increase in PM_2.5_ concentration is associated with over 30,000 deaths each year, equivalent to a loss of 0.13–0.15 years in national life expectancy [12].

The primary objective of epidemiology is to identify a causal connection between exposure and health outcome, thereby informing decisions on policy interventions [13]. For example, the United States Environmental Protection Agency (US EPA) reviews the National Ambient Air Quality Standards (NAAQS) periodically based on the cause-effect relationship that can be inferred from the best available science [14]. However, as observational studies, many air pollution epidemiological investigations, by nature, have been associational rather than causal [15]. Although a growing literature has examined the long-term effect of PM_2.5_ on mortality using the formal causal modeling techniques, there is so far little evidence for O_3_ and NO_2_ [16–18]. Indeed, O_3_ and NO_2_ have received less attention than they deserve; so far there is no standard for long-term O_3_ concentrations (only daily) and the standard for annual NO_2_ concentrations has remained the same for decades [19].

What has been mostly missing in air pollution epidemiology is the specific shapes of causal dose-response (D-R) relations between air pollution exposures and risk of mortality. Such causal D-R relations can provide profound implications in predicting the health impact at a target level of air pollution concentration [20]. Recently, a study of PM_10_ that explicitly used a formal causal modeling approach to estimate the D-R relationship found a higher mortality risk at low to moderate air pollution levels [21]. However, to date no such studies have been done for PM_2.5_, O_3_ or NO_2_. Specifying the causal D-R relationship, especially at very low levels, is critically important in measuring the risk of mortality induced directly by the change of air pollution level, thus supporting the potential revision of NAAQS in the US and, globally, the World Health Organization air quality guidelines [19, 22].

The present study analyzed 74 million Medicare beneficiaries in the contiguous US with 637 million person-years of follow-up from 2000 to 2016, which covers more than 95% of elders aged 65 years and older in the US who are considered to be most susceptible to air pollution [23]. The Medicare population also accounts for two-thirds of total mortality, allowing us to analyze most deaths induced by air pollution [1, 6]. By linking the annual averages of ambient PM_2.5_ and NO2 concentrations as well as warm-season (April–September) average of ambient O_3_ to the ZIP Codes of beneficiaries’ residence, we were able to have proxy measures of chronic exposures for each individual [24]. We proposed a decile binning approach which divided each exposure by deciles and predicted the inverse probability of being assigned to the observed group for each observation, adjusting for the other two concurrent exposures, personal characteristics, meteorological, socioeconomic, behavioral, and medical access variables, and long-term time trend. If propensity score models were correctly specified, we had constructed a valid counterfactual framework and thus estimated the causal D-R relations between chronic exposures to PM_2.5_, O_3_, and NO_2_ and the risk of all-cause mortality.

## Methods

### Mortality data

We obtained Medicare enrollment records for beneficiaries aged 65 years and above residing in the contiguous US between 2000 and 2016 from the Centers for Medicare and Medicaid Services, with all-cause mortality as the study outcome. For each beneficiary, we extracted their demographic information (sex, race, age at initial enrollment), Medicaid eligibility, ZIP Code of residence, year of initial enrollment, and year of death if it occurred during the study period. We constructed an open cohort with person-years of follow-up in which each beneficiary was followed each year from the study entry until the end of study, drop out of the cohort, or death, whichever occurred earliest. Note that the same data format has been used to fit time-varying Cox proportional hazard models [6].

### Exposure assessment

The daily concentrations of ambient PM_2.5_, O_3_, and NO_2_ at 1 km × 1 km grid cells across the contiguous US were predicted and validated using hybrid models that ensembled predictions from random forest, gradient boosting, and neural network. Multiple predictor variables were incorporated in the predictions, including ground monitoring data, satellite data, meteorological conditions, land-use variables, and chemical transport model simulations, etc., with details published elsewhere [25–27].

These high-resolution predictions at 1 km × 1 km grid cells allow us to estimated ZIP Code-level exposure levels with a high degree of accuracy, with annual R^2^ on held out monitors of 0.89 for PM_2.5_, 0.86 for O_3_, and 0.84 for NO_2_. There are two major types of ZIP Codes in the US: standard ZIP Code and PO Box. Because a standard ZIP Code represents a delivery area, we used the polygon layer generated by Environmental Systems Research Institute (Esri) [28], and estimated the ZIP Code’s daily concentrations by averaging the predictions at grid cells whose centroid points were inside the polygon of that ZIP Code. For PO Box, because it is used only for a given facility and therefore can be represented by a single point, we estimated its daily concentrations by linking it to the nearest grid cell.

The exposures of interest were assessed based on the ZIP Code-level estimates. For PM_2.5_ and NO_2_, we defined their chronic exposures as annual average concentrations. For O_3_, following previous literature [5, 6], we defined the chronic exposure as the average concentration during warm season (April–September) of the year. We assigned the chronic exposures to PM_2.5_, O_3_, and NO_2_ to each person-year based on that person’s ZIP Code of residence and calendar year.

### Covariate information

Meteorological variables including daily air temperature and humidity at 2 m above the ground were extracted from Phase 2 of the North American Land Data Assimilation System, with 12 km × 12 km resolution across the continental US [29]. The average temperatures during warm (April–September) and cold seasons (January–March plus October–December) of each year were calculated from the daily data because both exceedingly low and high temperatures were physically stressful and were also associated with air pollution levels [30, 31]. Annual average humidity was calculated from the daily data on a yearly basis. ZIP Code Tabulation Areas (ZCTA)-level socioeconomic variables, including the percentage of Blacks, percentage of Hispanics, median household income, median value of owner occupied housing, percentage of Americans aged 65 and older living below the poverty threshold, percentage of Americans with less than high school education, percentage of owner occupied housing units, and population density, etc., were obtained from 2000 and 2010 US Census and the American Community Survey [32]. These variables were linearly extrapolated by year to account for the time varying nature of socioeconomic status. County-level behavioral variables, including body mass index (BMI) and percentage of ever smokers for each year, were obtained from the Behavioral Risk Factor Surveillance System [33]. From the Dartmouth Atlas of Health Care [34], we obtained percentage of Medicare participants who had a hemoglobin A1c test, a low-density lipoprotein cholesterol (LDLC) test, a mammogram, and an eye exam to a primary care physician for each year in each hospital catchment area in the US and assigned it to all ZCTAs in that area. We also computed the distance from each ZIP Code centroid to the nearest hospital. These variables were linked to each person-year by ZIP Code of residence and calendar year. Summary statistics of the covariates are provided in Section 5 of Supplementary Information.

### A decile binning approach to emulate causal D-R relations

To emulate the causal D-R relationship, we need a counterfactual framework. For a binary exposure, the causal estimate in a population of interest comes from the difference between the counterfactual outcome under which all the members of the population had been exposed versus the counterfactual outcome had they not been exposed, thus no confounding occurs [35]. In randomized experiments, counterfactuals are constructed by randomly assigning individuals to treatment groups to ensure that exposure is independent of all potential confounders. In observational studies, however, the exposure assignment is not random but instead is considered to be influenced by subject characteristics, and causal methods seek ways to approximate counterfactuals with reference to the observed population [36]. Inverse probability weighting (IPW), for example, is a formal causal modeling technique and is increasingly being used in observational studies [37, 38]. For a binary exposure, it uses quasi-experimental design to construct a “pseudo-population” by weighting the population by the inverse probability of the observed exposure given all measured confounders. The “pseudo-population” is then used to estimate the exposure effect. If the systematic difference of characteristics among the exposed and unexposed is adequately adjusted so that the two groups are comparable with respect to any confounders, a causal conclusion is warranted [35, 39].

But air pollution exposure is continuous in nature. Estimating the inverse probability weights in the continuous setting is challenging as it needs to 1) correctly specify the distributional form of exposure, 2) deal with non-constant variance (heteroscedasticity), and 3) avoid excessively large or small weights for outliers that are more likely to occur [40, 41]. For these reasons, in this section we proposed a decile binning approach to emulate the causal D-R relations between chronic air pollution exposures and mortality by dividing each exposure into ten equal-sized groups by deciles, treating the lowest decile group (i.e., 10% of the study population with the lowest exposure levels) as the reference, and estimating the effects for the other groups compared to the reference. This relaxes the strong assumptions of distribution form and homoscedasticity for continuous exposure by relying solely on deciles. In addition, binning data makes the inverse probability weights robust against outliers [42].

We had a dataset with person-year representations of follow-up which allowed for time-varying exposures and covariates. To reduce the computational burden, first we aggregated the person-years with the same sex, race, age, Medicaid eligibility, living in the same ZIP Code of residence and in the same year. We treated them as a single record because those person-years had identical values for all exposures and covariates and thus could be treated interchangeably in the analysis. As a result, we retained all the information yet significantly reduced the size of the data in which each observation represented a stratum of combination of sex, race, age, and Medicaid eligibility per ZIP Code of residence per year. Numbers of deaths and person-years were cumulated for each stratum.

For each exposure, the analysis under the counterfactual framework was composed of two stages: a design stage where a randomized “pseudo-population” was constructed by weighting the observed population by the inverse probability of exposure given all measured confounders, and an analysis stage where the treatment effect was estimated among the constructed “pseudo-population” [43]. In the first stage, we binned the exposure into ten equal-sized categories based on deciles. The stabilized inverse probability weight (*sw*_*ij*_) for stratum *j* in exposure category *i* was defined as:

$$ {sw}_{ij}=\frac{P\left(X\in i\right)}{expit\left(g\left({X}_{ij};{n}_{ij}\ |\ \boldsymbol{C}\right)\right)} $$

where *P*(*X* ∈ *i*) denotes the probability of any observed exposure *X* being in group *i*, which equals to 0.1; *expit*(∙) denotes the inverse logistic link function where $expit(x)=\frac{\mathit{\exp}(x)}{1+\mathit{\exp}(x)}$; and *g*(∙) the gradient boosting machine (GBM) model with logistic loss function for predicting the probability of the observed categorized exposure *X*_*ij*_ given the set of confounders ***C***, weighted by *n*_*ij*_, the number of person-years aggregated in the stratum. The use of GBM for estimating the probability of observed exposure has demonstrated a better predictive accuracy compared to the conventional logistic regression, as it captures nonlinearity and interactions of confounders and is unaffected by the potential autocorrelation [44, 45]. The confounder set ***C*** includes the other two concurrent exposures, calendar year, the individual-level variables (sex, race, 5-year age group, and Medicaid eligibility), and the area-level meteorological, socioeconomic, behavioral, and medical access variables as detailed in the previous section. The numerator, *P*(*X* ∈ *i*), is used to stabilize the variability of weights to avoid excessively upweighting or downweighting observations [40].

In the second stage of the analysis, for each exposure, we fitted a log linear regression relating the number of deaths and factored exposure category, weighted by the stabilized inverse probabilities estimated from the first stage. We used quasi-Poisson link function to account for overdispersion in the number of death, and included an offset of the number of person-years to account for the different population size at risk in each stratum. As a result, we obtained the marginal effect of each decile group on mortality. If the model for estimating the stabilized inverse probability weight is correctly specified, we have achieved an unbiased estimator of the causal effect for each group [41].

The results are expressed as the relative risks (RR) of mortality for higher decile groups against the lowest-decile group (reference). The number of early deaths avoided by lowering air pollutant concentration of a higher to lower decile group can be calculated as $N{\alpha}_0\left(\frac{RR_{high}-1}{RR_{high}}-\frac{RR_{low}-1}{RR_{low}}\right)$, where *N* is the annual averaged number of person-years, *α*_0_ is the baseline annual mortality rate, and *RR*_*high*_ and *RR*_*low*_ are the relative risks of mortality for the higher decile group and the lower decile group, respectively. More details are provided in Section 4 of Supplementary Information.

We assessed the robustness of the causal dose-response relations between the chronic air pollution exposures and mortality risk by conducting sensitivity analysis of splitting each exposure into 14 bins.

## Results

The demographic characteristics of the national Medicare cohort during 2000–2016 were summarized in Table 1. The cohort included 74,537,533 Medicare beneficiaries with a total of 637,207,589 person-years of follow-up. The average follow-up time was 8.5 years. Among them 30,209,831 deaths occurred, accounting for 40.5% of the population. The cohort comprised more females (55.4%), mostly whites (84.0%), and mostly aged 65–74 years when entering the cohort (78.4%). Over 13 million beneficiaries ever enrolled in the Medicaid program, accounting for 18.5% of the population.

**Table 1:** Demographic characteristics of Medicare cohort, 2000–2016

Characteristics	No.	%
Population	74,537,533	100
Total person-years	637,207,589	
Average years of follow-up	8.5	
Death	30,209,831	40.5
Female	41,295,065	55.4
Race		
White	62,622,921	84.0
Black	6,638,467	8.9
Other	5,276,145	7.1
Age at cohort entry		
65–74	58,425,025	78.4
75–84	11,966,830	16.1
≥ 85	4,145,678	5.5
Enrollment in Medicaid	13,788,978	18.5

**Fig. 1:**
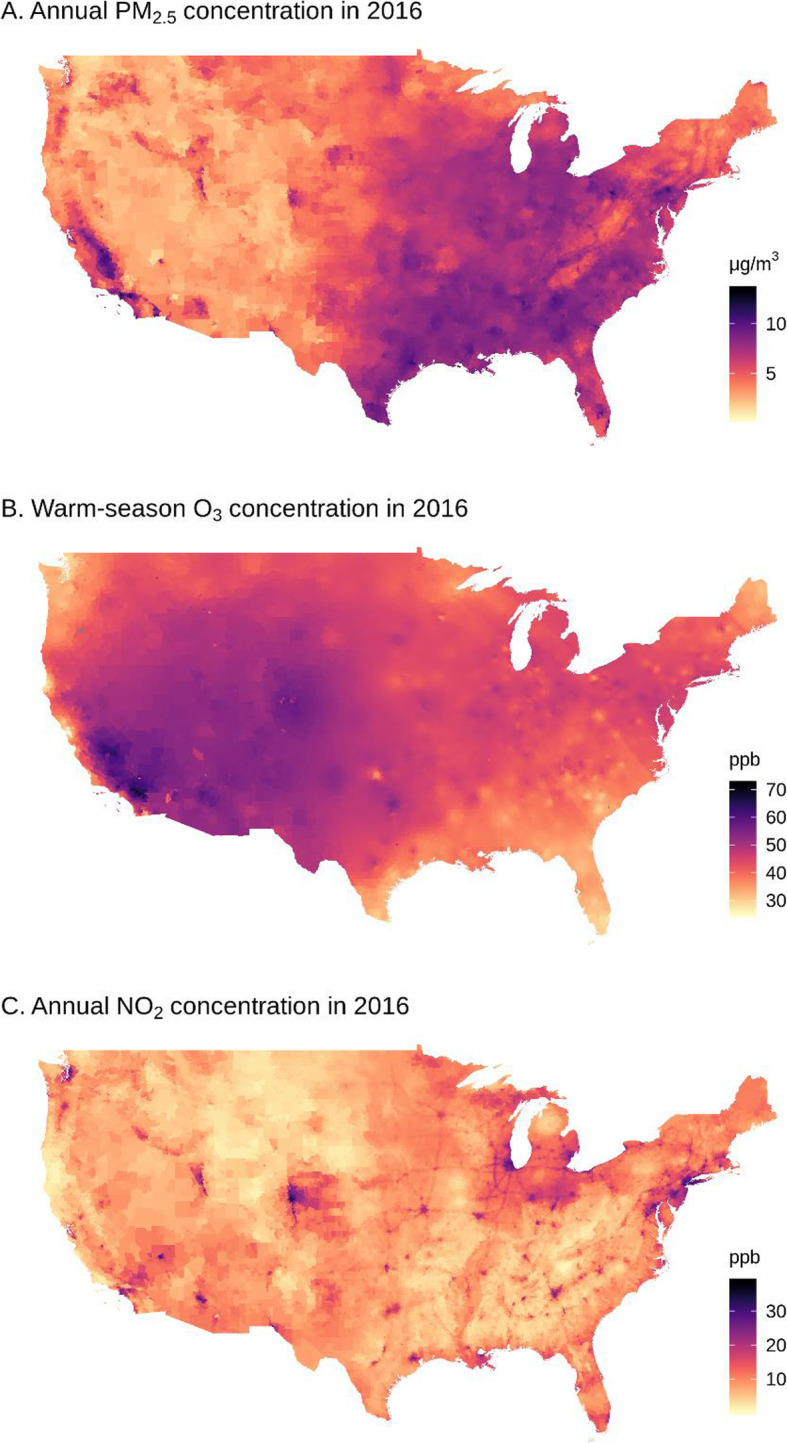
Maps of the contiguous US with annual PM_2.5_, warm-season O_3_, and annual NO_2_ concentrations at ZIP Code level in 2016

Maps of the contiguous US with annual PM_2.5_, warm-season (April–September) O_3_, and annual NO_2_ concentrations at ZIP Codes of the Medicare beneficiaries’ residence in 2016 are presented in Fig. 1. The PM_2.5_ concentration was higher in most central and eastern states and the Central Valley of California, and was lower in the northeast US and mountainous region. The warm-season O_3_ concentration was highest in the mountainous region and California. The NO_2_ concentration was higher in populous cities and along major highways. Over the years 2000–2016, the annual PM_2.5_ concentration at ZIP Codes averaged at 9.85 μg·m^− 3^, the warm-season O_3_ averaged at 39.34 ppb, and the annual NO_2_ averaged at 17.30 ppb (Table 2).

**Table 2:** Summary statistics for annual PM_2.5_, warm-season O_3_, and annual NO_2_ concentrations, 2000–2016

Statistics	PM_2.5_ (μg·m^−3^)	O_3_ (ppb)	NO_2_ (ppb)
Mean ± SD	9.85 ± 3.17	39.34 ± 4.03	17.30 ± 9.66
Decile			
10%	5.80	38.07	7.10
20%	7.28	40.97	9.09
30%	8.23	42.61	10.90
40%	9.00	43.86	12.83
50%	9.75	45.02	14.99
60%	10.53	46.38	17.55
70%	11.41	47.96	20.70
80%	12.46	49.93	24.93
90%	13.95	52.37	31.07

**Fig. 2:**
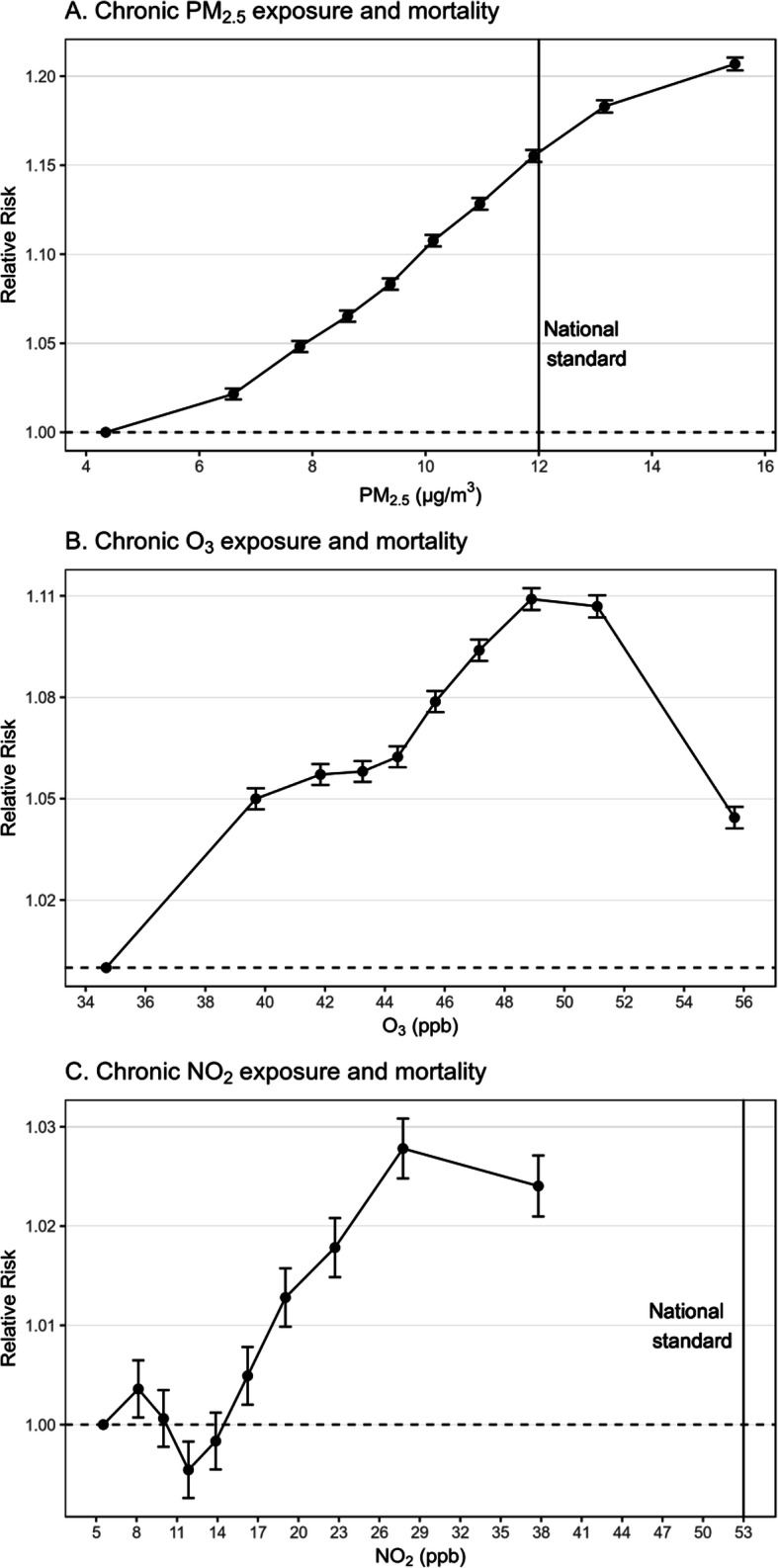
Causal dose-response relations between chronic exposures to PM_2.5_, O_3_, NO_2_ and the relative risk of mortality. The relative risks of mortality and 95% CIs are shown for higher decile groups against the lowest decile group (reference). The exposure concentration corresponding to each effect estimate represent the average concentration within the decile group. Vertical lines represent current national air quality standards for annual PM_2.5_ (12 μg·m^−3^) and annual NO_2_ (53 ppb). There is no long-term standard for O_3_

Figure 2 presents the estimated causal D-R relations between chronic exposures to PM_2.5_, O_3_, NO_2_ and the RR of mortality. The exposure concentration corresponding to each effect estimate represents the average concentration within the decile group. The dose-response relationship between chronic exposure to PM_2.5_ and mortality was monotonic and approximately linear, with higher concentration levels associated with greater risk of mortality. Specifically, the RRs of mortality associated with chronic exposure to PM_2.5_ ranged from 1.022 [95% confidence interval (CI), 1.018–1.025] at 6.60 μg·m^− 3^ (the 2nd decile group) to 1.207 (95% CI, 1.203–1.210) at 15.47 μg·m^− 3^ (the 10th decile group). For O_3_, the risk of mortality monotonically increased from the 2nd (RR, 1.050; 95% CI, 1.047–1.053) to the 9th decile group (RR, 1.107; 95% CI, 1.104–1.110), and dropped at the highest decile group (RR, 1.044; 95% CI, 1.041–1.048). For NO_2_, the dose-response curve wiggled at low levels and started rising from the 6th decile group (RR, 1.005; 95% CI, 1.002–1.018) till the highest decile group (RR, 1.024; 95% CI, 1.021–1.027). Importantly, the risk of mortality associated with chronic PM_2.5_ exposure was substantially larger than those with O_3_ and NO_2_; the highest RR for PM_2.5_ was greater than those for O_3_ and NO_2_. The entire dose-response relationship for NO_2_ occurred at concentrations below the national standard of 53 ppb, and most of the PM_2.5_ relationship was also below the standard of 12 μg·m^− 3^ [19]. There is no long-term standard for O_3_. All the numerical results are provided in Section 1 of Supplementary Information.

The dose-response relations remained robust after splitting each exposure into 14 bins, with details provided in Section 2 and Section 3 of Supplementary Information.

## Discussion

We proposed a decile binning approach to simultaneously emulate the D-R relations between chronic exposures to major air pollutants and mortality in a general and susceptible older population. Assuming that the IPW models were correctly specified and the counterfactual framework was valid, the D-R curves revealed that in general, higher levels of PM_2.5_, O_3_, and NO_2_ were causally associated with a greater risk of mortality. Compared with previous associational D-R curves [3, 5–7], the causal D-R curves essentially infer the number of potential lives saved if air pollution concentrations were reduced to targeting levels. For example, lowering each air pollutant concentration from the 70th to 60th percentiles would prevent 65,935 early deaths among elders per year (Section 4 of Supplementary Information), and this is a substantial public health benefit.

A major advance of the present study is that we simultaneously evaluated PM_2.5_, O_3_, and NO_2_, which allowed us to mutually adjust their confounding and also to directly compare their health impacts. We found that PM_2.5_ had a substantially larger effect on mortality than O_3_ and NO_2_. The finding confirmed previously published results suggesting that PM_2.5_ is the most deadly air pollutant and that chronic exposure to PM_2.5_ is of greater public health concern [18]. The increasing patterns of the D-R relations for PM_2.5_ and NO_2_ at levels below the current NAAQS suggest the necessity of more stringent national air quality standards for the protection of public health. Currently the NAAQS lack regulation for long-term O_3_, and clearly the daily standard has not reduced the warm-season average to concentrations with no mortality association [46]. Our results support the argument for establishing a warm-season O_3_ standard. The lower risk of mortality for the highest decile group for O_3_ may suggest that the O_3_ effect was represented by traffic exhausts such as nitrogen oxides and volatile organic compounds, as they play important roles in O_3_ actions and are highly reactive at extreme levels. However, further investigations are needed to address this question.

The causal conclusions of this study depend on the key assumption of correct IPW model specification. The validity of this assumption is not testable and relies on outside information. To minimize confounding bias, we adjusted for any known possible confounders such as concurrent air pollutants, Medicaid eligibility (proxy for individual’s low socioeconomic status), and seasonal temperatures and humidity (important physical stressors and determinants of air pollution [30]), etc. We also adjusted for area-level confounders of socioeconomic status, ethnicity, smoking status, obesity, population density, access to medical care, and calendar year (to capture unmeasured confounders that had a temporal scale of variation). In predicting the inverse probability of being assigned to the observed exposure decile given the set of confounders, GBM adaptively captured any nonlinearity and interactions and was unaffected by the potential autocorrelation [44]. Although residual confounding can never be ruled out, the consistent dose-response relationships for PM_2.5_ and O_3_ obtained across different study designs and populations provide some reassurance that our causal estimates are not substantially biased [3, 5–7].

As we have noted, the proposed decile binning approach relaxed assumptions on data distribution and homoscedasticity when constructing inverse probability weights for continuous exposures. Ambient air pollution concentrations usually follow a heteroscedastic distribution possibly with long tails, which results in excessively upweighted observations. To fix this issue, Naimi et al. proposed a quantile binning approach where he estimated weights for binned exposure and then treated those bins as continuous and linear, and found it outperformed other IPW estimators with various parametric forms of the exposure distribution [42]. Adopting his idea, our approach further relaxed the linear assumption by categorizing bins and comparing the effect of each bin to the reference group. If the assumption of correct IPW model specification holds, the estimated effect of each bin is an asymptotically unbiased estimator of the true causal effect [41]. Further, the estimand of our interest, the marginal effect estimates, do not depend upon the distributions of confounders and have arguably greater public health relevance because many confounders might not be measurable at decision time. The marginal effect estimates are also more useful when depicting dose-response relationship for the purpose of understanding the total effect [20].

Assigning ambient air pollution concentrations at ZIP Codes as a marker of individual exposure levels may result in measurement error. Although measuring more personal exposures can overcome the limitation, it also introduces confounding that are difficult to control such as personal behaviors, which may affect personal exposure measurement directly but not affect ambient air pollution estimation. In addition, personal exposure measurements can be compromised by the study outcome and thus is also more vulnerable to reverse causation [24]. For example, patients who die from chronic obstructive pulmonary disease (COPD) generally spent less time outdoors [47]; because ambient air pollutants are filtered by the building envelop and deposit on indoor surfaces, there are lower concentrations of those ambient pollutants indoors [48]. Hence, those patients have lower levels of personal exposures. By contrast, under the null assumption, COPD mortality is not associated with ambient concentration predictions, which are more proxy exposure measurement than the personal measurement. In epidemiology studies, ideally the measure of exposure should be as accurate as possible. In practice, however, this is usually not possible and the issue is to choose an appropriate exposure metric that balances the biological relevance, interpretability, and implications for public health policy. While using a proxy measure for air pollution exposure increases measurement error, it also brings important advantages for causal inference.

Some limitations must be acknowledged. First, we were not able to examine on cause-specific mortality which is not available for the Medicare data. Further studies investigating which major specific causes are driving the death would provide a valuable addition. Second, spatial confounding inherent to proximity-based air pollution measurements could still be present given that ZIP Code was the finest geographical unit we could use to link air pollution levels with each beneficiary. Third, restricted by available data sources, we could not adjust for individual behavior and medical history because such information was not available for the Medicare enrollment data, which may contribute to residual confounding. Fourth, although air pollution levels were estimated from models with excellent out-of-sample prediction ability, they are not perfect and therefore may attenuate effect estimates [24].

## Conclusions

In summary, this study simultaneously emulated D-R curves between chronic exposures to PM_2.5_, O_3_, NO_2_ and all-cause mortality among the national Medicare cohort during 2000–2016. We proposed a decile binning approach to relax the contentious assumptions of conventional IPW estimators, which yielded more robust causal evidence on adverse effects of air pollution exposure on mortality. Assuming that the IPW models were correctly specified, the estimated D-R curves reveal that in general, higher levels of chronic PM_2.5_, O_3_, and NO_2_ exposures were causally associated with a greater risk of mortality, even at levels below the national standards. Among the three pollutants, PM_2.5_ posed the greatest public health concern. The estimated D-R relations provide particularly significant implications for US EPA reviewing NAAQS, as the causal D-R curves essentially infer the number of potential lives saved if air pollution concentrations were reduced to specific levels. For example, lowering the air pollutant concentration from the 70th to 60th percentiles would prevent 65,935 early deaths among elders per year.

## Supplementary Information

**Additional file 1.**

## Data Availability

The exposure data are available from the corresponding author on reasonable request. The Medicare data are available upon request to the Centers for Medicare and Medicaid Services. The other data are publicly available, with sources described in the manuscript.

## Abbreviations

PM_2.5_: Ambient fine particulate matter
O_3_: Ozone
NO_2_: Nitrogen dioxide
US EPA: United States Environmental Protection Agency
NAAQS: National Ambient Air Quality Standards
D-R: Dose-response
IPW: Inverse probability weighting
ZCTA: ZIP Code Tabulation Areas
GBM: Gradient boosting machine
ppb: Parts per billion
COPD: Chronic obstructive pulmonary disease

## References

[1] Schraufnagel DE, Balmes JR, Cowl CT, De Matteis S, Jung SH, Mortimer K (2019). Air pollution and noncommunicable diseases: a review by the forum of international respiratory Societies’ environmental committee, part 2: air pollution and organ systems. Chest..

[2] Wei Y, Wang Y, Di Q, Choirat C, Wang Y, Koutrakis P (2019). Short term exposure to fine particulate matter and hospital admission risks and costs in the Medicare population: time stratified, case crossover study. BMJ..

[3] Dockery DW, Pope CA, Xu X, Spengler JD, Ware JH, Fay ME, Ferris BG, Speizer FE (1993). An association between air pollution and mortality in six U.S. cities. N Engl J Med.

[4] Brook RD, Rajagopalan S, Pope CA, Brook JR, Bhatnagar A, Diez-Roux AV (2010). Particulate matter air pollution and cardiovascular disease: an update to the scientific statement from the American Heart Association. Circulation..

[5] Jerrett M, Burnett RT, Pope CA, Ito K, Thurston G, Krewski D (2009). Long-term ozone exposure and mortality. N Engl J Med.

[6] Di Q, Dominici F, Schwartz JD (2017). Air pollution and mortality in the Medicare population. N Engl J Med.

[7] Burnett R, Chen H, Szyszkowicz M, Fann N, Hubbell B, Pope CA (2018). Global estimates of mortality associated with long-term exposure to outdoor fine particulate matter. Proc Natl Acad Sci U S A.

[8] Bowe B, Xie Y, Yan Y, Al-Aly Z (2019). Burden of Cause-Specific Mortality Associated With PM2.5 Air Pollution in the United States. JAMA Netw Open.

[9] Turner MC, Jerrett M, Pope CA, Krewski D, Gapstur SM, Diver WR (2016). Long-term ozone exposure and mortality in a large prospective study. Am J Respir Crit Care Med.

[10] Faustini A, Rapp R, Forastiere F (2014). Nitrogen dioxide and mortality: review and meta-analysis of long-term studies. Eur Respir J.

[11] Eum KD, Kazemiparkouhi F, Wang B, Manjourides J, Pun V, Pavlu V, Suh H (2019). Long-term NO2 exposures and cause-specific mortality in American older adults. Environ Int.

[12] Bennett JE, Tamura-Wicks H, Parks RM, Burnett RT, Pope CA, Bechle MJ (2019). Particulate matter air pollution and national and county life expectancy loss in the USA: a spatiotemporal analysis. PLoS Med.

[13] Lilienfeld DE (1978). Definitions of epidemiology. Am J Epidemiol.

[14] Owens EO, Patel MM, Kirrane E, Long TC, Brown J, Cote I, Ross MA, Dutton SJ (2017). Framework for assessing causality of air pollution-related health effects for reviews of the National Ambient air Quality Standards. Regul Toxicol Pharmacol.

[15] Hill AB (2015). The environment and disease: association or causation? 1965. J R Soc Med.

[16] Wang Y, Kloog I, Coull BA, Kosheleva A, Zanobetti A, Schwartz JD (2016). Estimating causal effects of Long-term PM2.5 exposure on mortality in New Jersey. Environ Health Perspect.

[17] Schwartz J, Bind MA, Koutrakis P (2017). Estimating causal effects of local air pollution on daily deaths: effect of low levels. Environ Health Perspect.

[18] Wei Y, Wang Y, Wu X, Di Q, Shi L, Koutrakis P (2020). Causal effects of air pollution on mortality in Massachusetts. Am J Epidemiol.

[19] U.S. E. 40 CFR Part 50 (1997). National ambient air quality standards for particulate matter: Final rule. Fed Regist.

[20] Cox LA (2017). Do causal concentration-response functions exist? A critical review of associational and causal relations between fine particulate matter and mortality. Crit Rev Toxicol.

[21] Forastiere L, Carugno M, Baccini M. Assessing short-term impact of PM10 on mortality using a semiparametric generalized propensity score approach. Environ Health. 2020;19(1):46. 10.1186/s12940-020-00599-6. 10.1186/s12940-020-00599-6 PMC7193397 32357874

[22] WHO. WHO Expert Consultation: Available Evidence for the Future Update of the WHO Global Air Quality Guidelines (AQGs). Geneva: WHO; 2016.

[23] Howden L, Meyer J. Age and sex composition: 2010 Census briefs: U.S. CENSUS BUREAU; 2011.

[24] Weisskopf MG, Webster TF (2017). Trade-offs of Personal Versus More Proxy Exposure Measures in Environmental Epidemiology. Epidemiology (Cambridge, Mass).

[25] Di Q, Amini H, Shi L, Kloog I, Silvern R, Kelly J (2019). An ensemble-based model of PM2.5 concentration across the contiguous United States with high spatiotemporal resolution. Environ Int.

[26] Requia WJ, Di Q, Silvern R, Kelly JT, Koutrakis P, Mickley LJ, Sulprizio MP, Amini H, Shi L, Schwartz J. An Ensemble Learning Approach for Estimating High Spatiotemporal Resolution of Ground-Level Ozone in the Contiguous United States. Environ Sci Technol. 2020;54(18):11037–47. 10.1021/acs.est.0c01791. 10.1021/acs.est.0c01791 PMC7498146 32808786

[27] Di Q, Amini H, Shi L, Kloog I, Silvern RF, Kelly JT (2019). Assessing NO2 concentration and model uncertainty with high spatiotemporal resolution across the contiguous United States using ensemble model averaging. Environ Sci Technol.

[28] Institute ESR (2010). Esri Data & Maps 10.

[29] Mitchell KE, et al. The multi-institution North American Land Data Assimilation System (NLDAS): Utilizing multiple GCIP products and partners in a continental distributed hydrological modeling system. J Geophys Res. 2004;109:D07S90. 10.1029/2003JD003823.

[30] Barreca AI (2012). Climate change, humidity, and mortality in the United States. J Environ Econ Manag.

[31] Shi L, Kloog I, Zanobetti A, Liu P, Schwartz JD (2015). Impacts of temperature and its variability on mortality in New England. Nat Clim Chang.

[32] Council NR (2007). Using the American community survey: benefits and challenges.

[33] CDC (2004). Behavioral Risk Factor Surveillance System Survey Questionnaire.

[34] Cronenwett JL, Birkmeyer JD (2000). The Dartmouth atlas of vascular health care. Cardiovasc Surg.

[35] Robins JM, Hernan MA, Brumback B (2000). Marginal structural models and causal inference in epidemiology. Epidemiology (Cambridge, Mass).

[36] Rosenbaum PR, Rubin DB (1983). The central role of the propensity score in observational studies for causal effects. Biometrika..

[37] Cole SR, Hernan MA (2008). Constructing inverse probability weights for marginal structural models. Am J Epidemiol.

[38] Hernan MA, Brumback B, Robins JM (2000). Marginal structural models to estimate the causal effect of zidovudine on the survival of HIV-positive men. Epidemiology (Cambridge, Mass).

[39] Hirano K, Imbens GW, Gelman A, Meng XL (2004). The Propensity Score with Continuous Treatments. Applied Bayesian Modeling and Causal Inference from Incomplete-Data.

[40] Robins J. Marginal structural models versus structural nested models as tools for causal inference. Stat Models Epidemiol Environment Clin Trials. 2000:95–133. 10.1007/978-1-4612-1284-3_2.

[41] Hernan MA, Robins JM (2006). Estimating causal effects from epidemiological data. J Epidemiol Community Health.

[42] Naimi AI, Moodie EE, Auger N, Kaufman JS (2014). Constructing inverse probability weights for continuous exposures: a comparison of methods. Epidemiology (Cambridge, Mass).

[43] Rubin DB (2008). For objective causal inference, design trumps analysis. Ann Appl Stat.

[44] McCaffrey DF, Ridgeway G, Morral AR (2004). Propensity score estimation with boosted regression for evaluating causal effects in observational studies. Psychol Methods.

[45] Friedman J. Greedy function approximation: a gradient boosting machine. Ann Stat. 2011:1189–232.

[46] Di Q, Dai L, Wang Y, Zanobetti A, Choirat C, Schwartz JD (2017). Association of Short-term Exposure to air pollution with mortality in older adults. JAMA..

[47] Donaldson GC, Wilkinson TM, Hurst JR, Perera WR, Wedzicha JA (2005). Exacerbations and time spent outdoors in chronic obstructive pulmonary disease. Am J Respir Crit Care Med.

[48] DYC L. Outdoor-indoor air pollution in urban environment: challenges and opportunity. Front Environ Sci. 2015;2:69. 10.3389/fenvs.2014.00069.

