# Supplementary material for: Emulating causal dose-response relations between air pollutants and mortality in the Medicare population
Source: Environ Health. 2021 May 6;20:53. doi: 10.1186/s12940-021-00742-x (PMC8103595; doi:10.1186/s12940-021-00742-x)
Supplement: Supplementary file 1 — Additional file 1. [file 12940_2021_742_MOESM1_ESM.docx]

**Supplementary Information**

*for*

**Causal dose-response relations between air pollutants and mortality in elders**

**Section 1. Numerical results of main analysis.**

**Section 2. Numerical results of the sensitivity analysis with respect to splitting exposure into 14 bins.**

**Section 3. Dose-response results of the sensitivity analysis with respect to splitting exposure into 14 bins.**

**Section 4. Calculation of the number of early deaths avoided by lowering an air pollutant concentration of a higher decile group to a lower decile group.**

**Section 5. Descriptive statistics of community-level covariates calculated from study cohort, 2000–2016.**

**Section 1. Numerical results of main analysis.**

| **Exposure** | **Decile group** | **Mean concentration** | **RR of mortality** | **95% CI** |
| --- | --- | --- | --- | --- |
| PM_2.5_ (µg·m^-3^) | 1 (reference) | 4.35 | 1 | NA |
|  | 2 | 6.60 | 1.022 | (1.018, 1.025) |
|  | 3 | 7.78 | 1.048 | (1.045, 1.051) |
|  | 4 | 8.62 | 1.065 | (1.062, 1.068) |
|  | 5 | 9.38 | 1.083 | (1.080, 1.086) |
|  | 6 | 10.13 | 1.108 | (1.104, 1.111) |
|  | 7 | 10.96 | 1.128 | (1.125, 1.132) |
|  | 8 | 11.91 | 1.155 | (1.152, 1.159) |
|  | 9 | 13.16 | 1.183 | (1.180, 1.186) |
|  | 10 | 15.47 | 1.207 | (1.203, 1.210) |
| O_3_ (ppb) | 1 (reference) | 34.69 | 1 | NA |
|  | 2 | 39.69 | 1.050 | (1.047, 1.053) |
|  | 3 | 41.85 | 1.057 | (1.054, 1.060) |
|  | 4 | 43.26 | 1.058 | (1.055, 1.061) |
|  | 5 | 44.43 | 1.062 | (1.059, 1.065) |
|  | 6 | 45.69 | 1.078 | (1.076, 1.082) |
|  | 7 | 47.15 | 1.094 | (1.091, 1.097) |
|  | 8 | 48.90 | 1.109 | (1.106, 1.112) |
|  | 9 | 51.10 | 1.107 | (1.104, 1.110) |
|  | 10 | 55.69 | 1.044 | (1.041, 1.048) |
| NO_2_ (ppb) | 1 (reference) | 5.53 | 1 | NA |
|  | 2 | 8.13 | 1.004 | (1.001, 1.006) |
|  | 3 | 10.00 | 1.001 | (0.998, 1.003) |
|  | 4 | 11.84 | 0.995 | (0.993, 0.998) |
|  | 5 | 13.87 | 0.998 | (0.995, 1.001) |
|  | 6 | 16.23 | 1.005 | (1.002, 1.008) |
|  | 7 | 19.04 | 1.013 | (1.010, 1.016) |
|  | 8 | 22.71 | 1.018 | (1.015, 1.021) |
|  | 9 | 27.75 | 1.028 | (1.025, 1.031) |
|  | 10 | 37.79 | 1.024 | (1.021, 1.027) |

**Section 2. Numerical results of the sensitivity analysis with respect to splitting exposure into 14 bins.**

| **Exposure** | **Decile group** | **Mean concentration** | **RR of mortality** | **95% CI** |
| --- | --- | --- | --- | --- |
| PM_2.5_ (µg·m^-3^) | 1 (reference) | 3.82 | 1 | NA |
|  | 2 | 5.77 | 1.018 | (1.014, 1.021) |
|  | 3 | 6.87 | 1.029 | (1.026, 1.033) |
|  | 4 | 7.66 | 1.051 | (1.047, 1.055) |
|  | 5 | 8.25 | 1.060 | (1.056, 1.064) |
|  | 6 | 8.75 | 1.073 | (1.069, 1.077) |
|  | 7 | 9.30 | 1.085 | (1.082, 1.089) |
|  | 8 | 9.85 | 1.104 | (1.100, 1.108) |
|  | 9 | 10.39 | 1.120 | (1.116, 1.124) |
|  | 10 | 10.99 | 1.135 | (1.131, 1.139) |
|  | 11 | 11.64 | 1.154 | (1.150, 1.158) |
|  | 12 | 12.43 | 1.173 | (1.169, 1.177) |
|  | 13 | 13.43 | 1.192 | (1.187, 1.196) |
|  | 14 | 15.51 | 1.213 | (1.209, 1.217) |
| O_3_ (ppb) | 1 (reference) | 33.69 | 1 | NA |
|  | 2 | 38.37 | 1.038 | (1.034, 1.041) |
|  | 3 | 40.52 | 1.064 | (1.060, 1.067) |
|  | 4 | 41.88 | 1.063 | (1.059, 1.066) |
|  | 5 | 42.86 | 1.063 | (1.060, 1.067) |
|  | 6 | 43.71 | 1.064 | (1.060, 1.067) |
|  | 7 | 44.49 | 1.068 | (1.064, 1.072) |
|  | 8 | 45.34 | 1.081 | (1.077, 1.084) |
|  | 9 | 46.24 | 1.090 | (1.086, 1.094) |
|  | 10 | 47.24 | 1.101 | (1.097, 1.105) |
|  | 11 | 48.43 | 1.112 | (1.108, 1.116) |
|  | 12 | 49.82 | 1.120 | (1.116, 1.124) |
|  | 13 | 51.51 | 1.109 | (1.105, 1.113) |
|  | 14 | 55.83 | 1.048 | (1.045, 1.052) |
| NO_2_ (ppb) | 1 (reference) | 4.89 | 1 | NA |
|  | 2 | 6.99 | 1.006 | (1.002, 1.010) |
|  | 3 | 8.34 | 1.004 | (1.001, 1.008) |
|  | 4 | 9.55 | 1.001 | (0.998, 1.005) |
|  | 5 | 10.74 | 1.003 | (1.000, 1.007) |
|  | 6 | 12.00 | 0.995 | (0.992, 0.999) |
|  | 7 | 13.38 | 0.998 | (0.995, 1.002) |
|  | 8 | 14.96 | 1.003 | (0.999, 1.007) |
|  | 9 | 16.82 | 1.006 | (1.003, 1.010) |
|  | 10 | 19.00 | 1.014 | (1.011, 1.018) |
|  | 11 | 21.78 | 1.016 | (1.012, 1.019) |
|  | 12 | 25.31 | 1.025 | (1.021, 1.028) |
|  | 13 | 30.08 | 1.029 | (1.025, 1.032) |
|  | 14 | 39.58 | 1.026 | (1.022, 1.029) |

**Section 3. Dose-response results of the sensitivity analysis with respect to splitting exposure into 14 bins.**


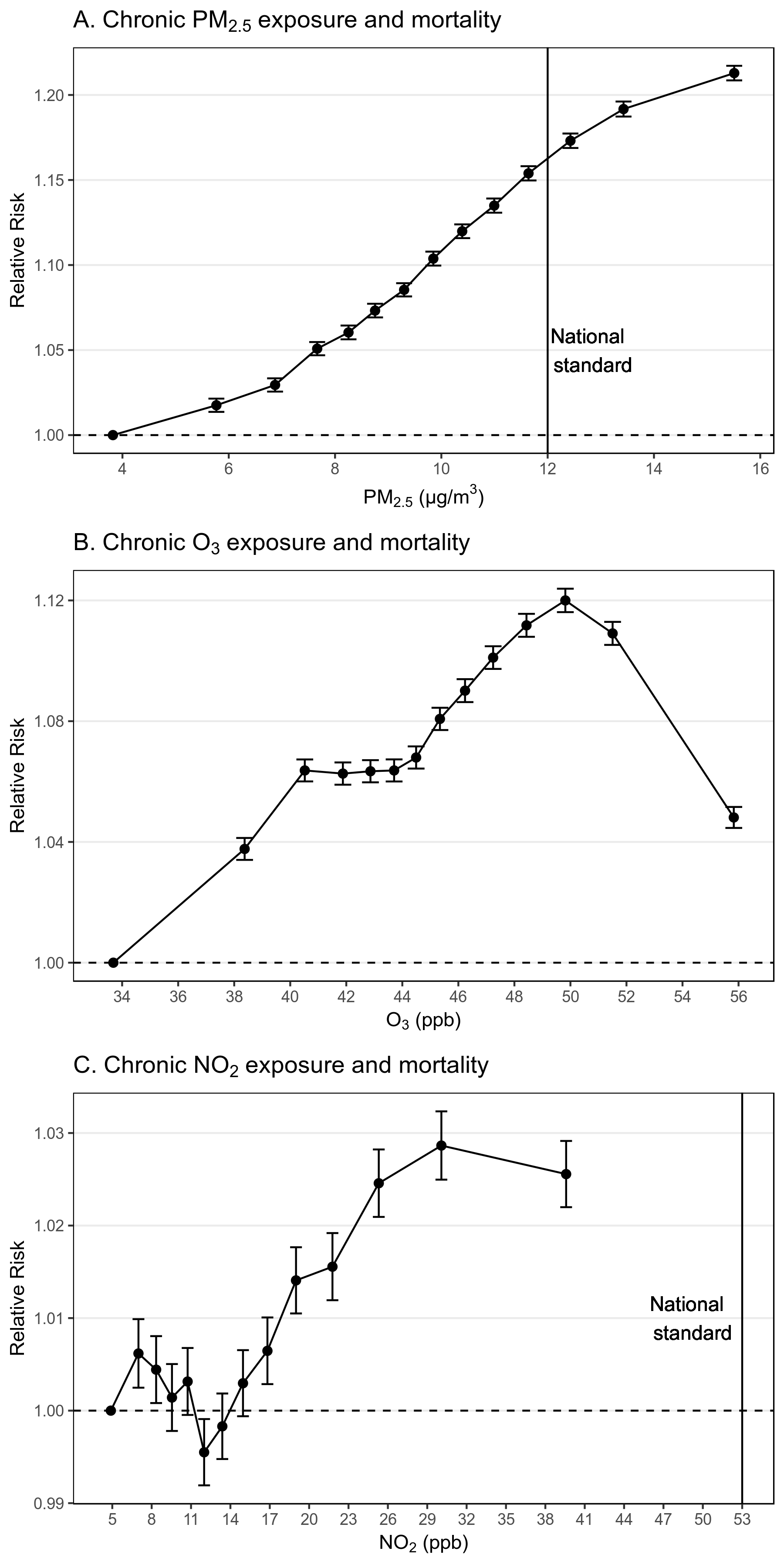


**Section 4. Calculation of the number of early deaths avoided by lowering an air pollutant concentration of a higher decile group to a lower decile group.**

To calculate the number of early deaths avoided by lowering an air pollutant concentration of a higher decile group to the concentration of a lower decile group, we first calculated the baseline annual mortality rate ($\alpha_{0}$) using the total number of deaths during 2000–2016 among the Medicare cohort divided by the total number of person-years, which was the sum of number of persons at risk in each year during 2000–2016. The baseline annual mortality rate $\alpha_{0}=0.047$.

According to the delta method, we then calculated the number of early deaths avoided by lowering an air pollutant concentration of a higher decile group to a lower decile group as $N\alpha_{0}\left( \frac{{RR}_{high}-1}{{RR}_{high}}-\frac{{RR}_{low}-1}{{RR}_{low}} \right)$, where $N$ is the annual averaged number of person-years, and ${RR}_{high}$ and ${RR}_{low}$ are the relative risks of mortality for the higher decile group and the lower decile group, respectively. For example, the number of early deaths avoided by lowering each air pollutant concentrations from the 70th to 60th percentiles can be calculated as:

$N\alpha_{0}\left( \frac{{RR}_{70th}^{{PM}_{2.5}}-1}{{RR}_{70th}^{{PM}_{2.5}}}-\frac{{RR}_{60th}^{{PM}_{2.5}}-1}{{RR}_{60th}^{{PM}_{2.5}}} \right)+N\alpha_{0}\left( \frac{{RR}_{70th}^{O_{3}}-1}{{RR}_{70th}^{O_{3}}}-\frac{{RR}_{60th}^{O_{3}}-1}{{RR}_{60th}^{O_{3}}} \right)+N\alpha_{0}\left( \frac{{RR}_{70th}^{{NO}_{2}}-1}{{RR}_{70th}^{{NO}_{2}}}-\frac{{RR}_{60th}^{{NO}_{2}}-1}{{RR}_{60th}^{{NO}_{2}}} \right)=65,935$.

This method is consistent with Di et al. (1) and Wei et al. (2)

**Section 5. Descriptive statistics of community-level covariates calculated from study cohort, 2000–2016.**

| **Community-level covariate** | **Mean** | **SD** |
| --- | --- | --- |
| Mean temperature in warm season (April-September) (°C) | 18.6 | 4.2 |
| Mean temperature in cold season (January-March plus October-December) (°C) | 0.7 | 8.2 |
| Specific humidity of a kilogram of air (g/kg) | 6.6 | 2.4 |
| Median household income (USD) | 58,177 | 22,082 |
| Median house value (USD) | 200,139 | 159,728 |
| Percent of Blacks | 10.4% | 17.9% |
| Percent of Hispanic | 12.6% | 16.4% |
| Percent ≥ 65 years of age below poverty | 9.5% | 6.5% |
| Percent of low education | 25.3% | 14.7% |
| Percent with annual mammogram | 68.7% | 7.2% |
| Percent with ambulatory visit | 77.8% | 6.2% |
| Population density (persons/mi^2^) | 3,397 | 9,032 |
| Percent of owner-occupied units | 68% | 16% |
| Mean BMI (kg/m^2^) | 27.5 | 1.6 |
| Distance to nearest hospital (km) | 6.5 | 7.4 |
| Percent of ever smokers | 46.2% | 6.8% |
| Percent of annual HbA1c test | 33.1% | 4.9% |
| Lung cancer rate (×10^4^) | 3.9 | 2.8 |
| Percent of annual LDL test | 79.5% | 6.2% |
| Percent of annual eye exam | 67.4% | 6.4% |

**References**

1. Di Q, Dai L, Wang Y, Zanobetti A, Choirat C, Schwartz JD, et al. Association of Short-term Exposure to Air Pollution With Mortality in Older Adults. Jama. 2017;318(24):2446-56.

2. Wei Y, Wang Y, Wu X, Di Q, Shi L, Koutrakis P, et al. Causal Effects of Air Pollution on Mortality in Massachusetts. American journal of epidemiology. 2020.
